# Central retinal vein occlusion: a case report and review of the literature

**DOI:** 10.1186/1757-1626-2-7170

**Published:** 2009-06-03

**Authors:** Tarek Alasil, Nahyoung Lee, Pearse Keane, Srinivas Sadda

**Affiliations:** Doheny Eye Institute, University of Southern CaliforniaLos Angeles, CA 90033USA

## Abstract

**Introduction:**

Central retinal vein occlusion is one of the major causes of severe vision impairment and blindness in adults.

**Case presentation:**

We present a case of unilateral ischemic central retinal vein occlusion in a 54-year-old woman with history of uncontrolled hypertension and open angle glaucoma. Laboratory tests including complete hypercoagulability and thrombotic workup were completed.

**Conclusion:**

Our case illustrates an interesting presentation of unilateral ischemic central retinal vein occlusion, where hypertension and glaucoma were thought to be the main risk factors. Close follow up, tight blood pressure and glaucoma control are crucial to prevent similar scenario in the fellow eye.

## Introduction

Central retinal vein occlusion (CRVO) is one of the major causes of severe vision impairment and blindness [1].

Thrombosis of the central retinal vein results in venous stasis, leading to disc swelling, diffuse nerve fiber layer and pre-retinal hemorrhage, and cotton wool spots that create a dramatic appearance, often called “the blood and thunder” fundus.

The prevalence of central retinal vein occlusion (CRVO) is 0.1 to 0.7 percent in population-based studies [2]. A population-based study found a cumulative incidence of 0.5 percent over 15 years, with a prevalence of 1.3 percent for people aged 65 years and older [3].

Occlusion or thrombosis of the central retinal vein is associated with chronic glaucoma, atherosclerotic risk factors (age, diabetes, and hypertension), hyperviscosity, coagulopathy, and migraine [3-6]. The cause of retinal vein occlusion is often unknown.

While vision loss may be severe, the onset is typically subacute, in contrast to the sudden visual loss typical of central retinal artery occlusion. When venous stasis is severe, infarction may occur due to slowed retinal blood flow on the arterial side. In this setting, a relative afferent papillary defect is often present.

We present an interesting case of unilateral ischemic central retinal vein occlusion. We will discuss the risk factors, the complications, and the management guidelines in CRVO.

## Case presentation

A 54-year-old African American female with history of uncontrolled hypertension and glaucoma presented to the eye clinic with sudden vision loss in the right eye upon awakening. She has been taking Clonidine 10 mg oral twice daily and Latanoprost 1 drop in each eye twice daily.

Vital signs upon presentation are summarized in Table 1. Electrocardiogram showed sinus rhythm, and left ventricular hypertrophy.

**Table 1.** Vital signs upon presentation are summarized in Table 1

Temperature	97.0 F
Respiratory rate	19 breath/min
Blood pressure	190/103 mmHg
Heart rate	64 bpm
Pain	0/10

Ophthalmologic exam showed best corrected visual acuity of hand motion in the right eye and 20/70 in the left eye. Pupil exam showed sluggish right pupil with relative afferent papillary defect, and a reactive pupil on the left.

Intraocular pressures were 19 mmHg in the right, and 11 mmHg in the left.

Slit lamp exam showed normal anterior segments with open angles bilaterally.

**Figure 1.**
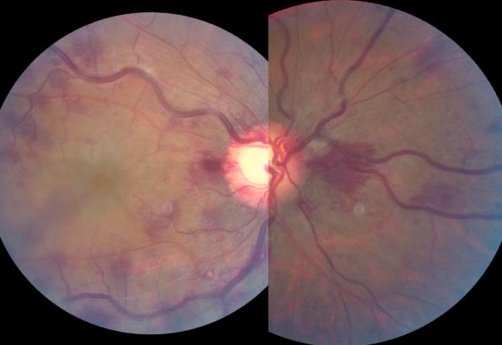
Color Fundus photograph of the right eye showed superficial opacification of the retina in the macular area in combination with multiple dot-blot and flame-shaped hemorrhages in all four quadrants (including the area nasally to the optic nerve).

Fundus examination of the right eye revealed cup to disk ratio of 0.7, dilated and tortuous retinal veins, with intraretinal hemorrhages especially in the peripapillary region (Figure 1). Fundus examination of the left eye revealed cup to disk ratio of 0.7, otherwise normal optic disc and flat macula.

Differential diagnosis in the right eye included central retinal vein occlusion (CRVO), ocular ischemic syndrome, diabetic retinopathy, papilledema, and acute hypertensive retinopathy (Figure 3).

**Figure 2.**
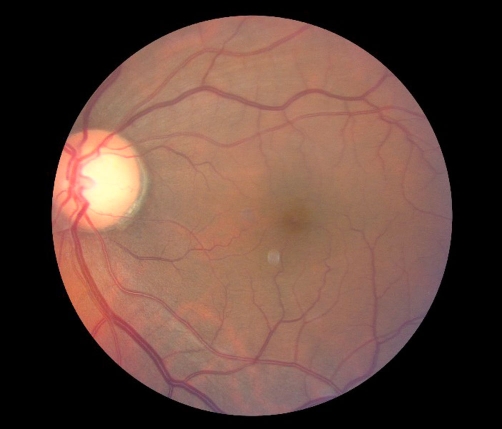
Color Fundus photograph of the left eye showed normal optic disc, and flat macula (normal findings).

**Figure 3.**
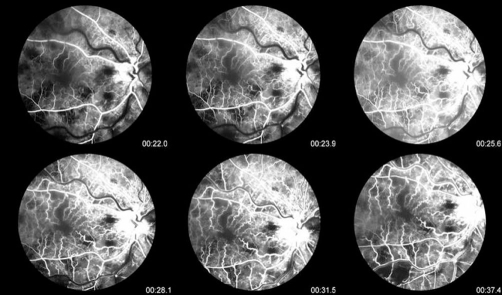
Flourescein Angiogram of the right eye showed blocked venous fluorescence from the retinal hemorrhages, extensive areas of capillary non-perfusion, and vessel wall staining.

**Figure 4.**
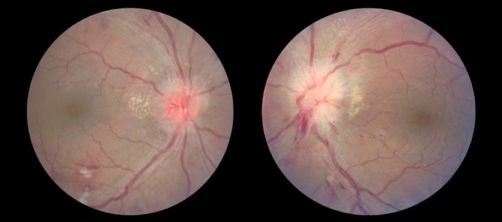
An example of acute hypertensive retinopathy, which is one of the differentials for CRVO. Figures here are showing arteriovenous nicking, copper wire arterial changes, hemorrhages, cotton wool spots, disc edema bilaterally (Left more than the right), and exudates that dominate in the peripapillary area.

Flourescein Angiogram of the right eye showed blocked venous fluorescence from the retinal hemorrhages, extensive areas of capillary non-perfusion, and vessel wall staining. Patient was diagnosed with unilateral ischemic central retinal vein occlusion in the right eye. Laboratory tests including complete hypercoagulability and thrombotic workup were completed (Tables 2, 3).

**Table 2.** Laboratory tests including hypercoagulability workup are summarized in Table 2

Glucose	90
HgA1c	5.8
White blood cell count	9.8
Neutrophils	80%
Lymphocytes	14%
Monocytes	4.6%
Hemoglobin	15.0
Hematocrit	45.1
Platelets	282
MCV	84.3
RDW	14.6
Prothrombin time	12.5
INR	0.99
PTT LA	33 normal (Reference normal < 40)
ANA	Negative
Rheumatoid factor	Negative
ESR	10 (normal)
CRP	20 (normal)
Serum protein electrophoresis	Normal
Lipid profile	Normal
Hemoglobin electrophoresis	Normal
VDRL	Negative
FTA-ABS	Negative

**Table 3.** Further hypercoagulability workup is summarized in Table 3

HIV	Negative
Functional protein S assay	Normal
Functional protein C assay	Normal
Functional antithrombin III assay	Normal
Antiphospholipid antibody titer	262 (reference range = 151-264)
Lupus anticoagulant	Negative
Anticardiolipin antibody	Negative
Homocysteine	11.5 (0-10) High
Folate level	Normal
B12 level	Normal
Creatinine	0.9 mg per deciliter
Factor V Leiden PCR assay	Negative

Patient was prescribed aspirin 81 mg daily. Amlodipine 10 mg, and Hydrochlorothiazide 25 mg were added to achieve better blood pressure control. Timolol and Brimonide eye drops were added to achieve better control of the intraocular pressure.

Patient was advised to follow closely during the next six months, including gonioscopy and undilated examination of the iris to check for neovascularization of the iris/disc.

## Discussion

CRVO has two types:

- Nonischemic (70%): which is characterized by vision that is better than 20/200, 16% progress to nonperfused; 50% resolve completely without treatment; defined as <10 disk diameter (DD) of capillary nonperfusion.
- Ischemic (30%): which is defined as more than 10 DD of nonperfusion; patients are usually older and have worse vision; 60% develop iris NV; up to 33% develop neovascular glaucoma; 10% are combined with branch retinal arterial occlusion (usually cilioretinal artery due to low perfusion pressure of choroidal system) [7].

Central retinal vein occlusion is a disease of the old population (age >50 years old). Major risk factors are hypertension, diabetes, and atherosclerosis. Other risk factors are glaucoma, syphilis, sarcoidosis, vasculitis, increased intraorbital or intraocular pressure, hyphema, hyperviscosity syndromes (multiple myeloma, Waldenstrom's macroglobulinemia, and leukemia), high homocysteine levels, sickle cell, and HIV [8].

Paul O'Mahoney et al. studied the relationship between traditional atherosclerosis risk factors and retinal vein occlusion (RVO). They systematically retrieved all studies between 1985 and 2007 that compared cases with any RVO with controls. They concluded that hypertension and hyperlipidemia are common risk factors for RVO in adults, and diabetes mellitus is less so. It remains to be determined whether lowering blood pressure and/or serum lipids levels can improve visual acuity or the complications of RVO [9].

Open-angle glaucoma is the most common local factor predisposing to RVO as increased intraocular pressure compromises retinal vein outflow and produces stasis [10,11].

Every eye with CRVO is at risk for developing neovascular glaucoma. Lowering intraocular pressure helps to improve retinal circulation in an eye with CRVO [12], and there is a 10% risk for development of BRVO or CRVO in the fellow eye [13].

Risk factors for developing neovascular iris in patients with CRVO are the amount of nonperfused retina, extent of retinal hemorrhages, male sex, and central vein occlusion of less than one month duration [14]. Visual acuity in patients with CRVO at baseline is a strong predictor for the development of INV/ANV, as is the amount of nonperfusion seen by fluorescein angiogram [1].

The central vein occlusion study (CVOS) data did not support the recommendation for prophylactic panretinal photocoagulation (PRP). The CVOS found that early PRP decreased the rate of iris neovascularization (INV); however, the reduction was not statistically significant. Moreover, the study showed that early PRP reduced, but did not eliminate, the possibility of anterior-segment neovascularization. The CVOS recommended close follow-up of eyes with CRVO during the first 6 months (including gonioscopy and undilated slit lamp examination of the iris) and prompt PRP of eyes in which iris neovascularization (INV)/Angle neovascularization (ANV) develops [14].

Although PRP was better than selective PRP or photodynamic therapy (PDT) at determining INV and anterior segment neovascularization regression, selective PRP or PDT can also be safely used to manage anterior segment neovascularization secondary to ischemic CRVO [15].

## Conclusion

Our case illustrates an interesting presentation of unilateral ischemic central retinal vein occlusion, where hypertension and glaucoma were thought to be the main risk factors. While many interventions for fixed visual loss associated with CRVO have largely not proven to be of benefit, our management focused on controlling blood pressure and glaucoma as preventive measures to protect the fellow eye. Meanwhile, close follow up was emphasized to investigate for signs of iris/angle neovascularization.

## Abbreviation

**List of abbreviatons**
ANV: Angle neovascularization
CRVO: Central retinal vein occlusion
CVOS: Central vein occlusion study
INV: Iris neovascularization
PDT: Photodynamic therapy
PRP: Panretinal photocoagulation
